# Acceleration of somatic cell reprogramming into the induced pluripotent stem cell using a mycosporine-like amino acid, Porphyra 334

**DOI:** 10.1038/s41598-020-60680-5

**Published:** 2020-02-28

**Authors:** Junsang Yoo, Junyeop Kim, Jeong Hun Lee, Hyein Kim, Sung Joo Jang, Hyo Hyun Seo, Seung Taek Oh, Seung Jae Hyeon, Hoon Ryu, Jongpil Kim, Sang Hyun Moh

**Affiliations:** 1 Anti-aging Research Institute, BIO-FD&C Co., Ltd, Inchon, 21990 Korea; 2 Center for Neuroscience, Brain Science Institute, Korea Institute of Science and Technology, Seoul, 02792 Korea; 3 Department of Biomedical Engineering, Dongguk University, Seoul, 100‐715 Korea

**Keywords:** Reprogramming, Stem-cell differentiation

## Abstract

Porphyra 334 (P334), a mycosporine-like amino acid (MAA), is a secondary metabolite found in diverse marine and terrestrial organisms and has several beneficial effects on fibroblast proliferation, wound healing, and antioxidant activity. Here, we report that P334 accelerates the cell reprogramming process of mouse tail-tip fibroblasts (TTFs) and human dermal papilla (HDP) cells into induced pluripotent stem cells (iPSCs). We found that P334 significantly improved the cell reprogramming efficiency by activating the tri-methylation of histone 3 lysine 4 (H3K4me3), which controls mesenchymal to epithelial transition (MET) during the reprogramming process. Thus, we found that P334 directly regulates epigenetic changes, providing an efficient approach for natural compound-based cell reprogramming.

## Introduction

Recently, natural products from marine organisms have gained a large amount of interest in the regenerative medicine field^1–3^. Among these natural products is porphyra 334 (P334), a member of the family of mycosporine-like amino acids (MAAs), which are natural compounds found in a variety of organisms, including fungi, bacteria, cyanobacteria, phytoplankton, and macro-algae^4–6^. P334 has received much attention because of its functional roles in ultraviolet (UV) protection^7–9^ and ROS scavenging (antioxidant activity)^10–13^. Furthermore, one of its unique characteristics is that it is highly resistant to abiotic stressors, such as temperature, various solvents, and pH^14^. Additionally, other characteristics of P334 have been reported to activate focal adhesion kinases (FAKs), extracellular signal-regulated kinase (ERK), and c-Jun N-terminal kinases (JNKs), which are essential for the wound healing processes^15^. Because of these unique characteristics, we examined the use of this compound in cell fate conversion through epigenetic reprogramming.

Cell reprogramming into pluripotency involves the generation of embryonic stem cell (ESC)-like cells from somatic cells via the ectopic expression of defined transcriptional factors^16,17^. Yamanaka demonstrated that the ectopic expression of four transcription factors (Oct4, Klf4, Sox2, and c-Myc (OSKM)) could induce the conversion of murine somatic cells to induced pluripotent stem cells (iPSCs)^18^. Moreover, studies using iPSCs provide a unique experimental approach to investigate key questions regarding cell fate conversion and epigenetic changes following the induction of pluripotency marker genes and the suppression of somatic cell genetic characteristics.

However, iPSC generation has a relatively low efficiency and slow kinetics (only 1–20% of iPSCs express the endogenous pluripotency markers after 3–4 weeks) and is considered a stochastic process^17,19^. Particularly, one of the most noticeable changes in the reprogramming process is the morphological transformation of cells into clusters of shiny, round cells, resembling mesenchymal to epithelial transition (MET)^20^. During the developmental process, epithelial to mesenchymal transition (EMT) occurs as early as gastrulation. Cell-to-cell and cell-to-matrix interactions are involved in the EMT process and lead to the loss of epithelial characteristics and the acquisition of mesenchymal lineage markers by downregulating E-cadherin (Cdh1) and upregulating Snail and Slug. Specifically, Cdh1 is a transmembrane constituent of intercellular adherens junctions, which are responsible for maintaining epithelial cohesion, and plays a key role in controlling ESC pluripotency and somatic cell reprogramming^21,22^.

In this study, we showed that P334 not only leads to efficient cell reprogramming via the activation of MET but also highly activates the tri-methylation of histone 3 lysine 4 (H3K4me3) in P334-mediated cell reprogramming, which eventually leads to a higher reprogramming efficiency^23–27^. Consequently, our finding supports a model in which the natural compound P334 affects the epigenetic state associated with cell fate conversion, which has important implications for the application of P334 in stem cell biology research and regenerative medicine.

## Results

### Characterization of P334

**Figure 1:**
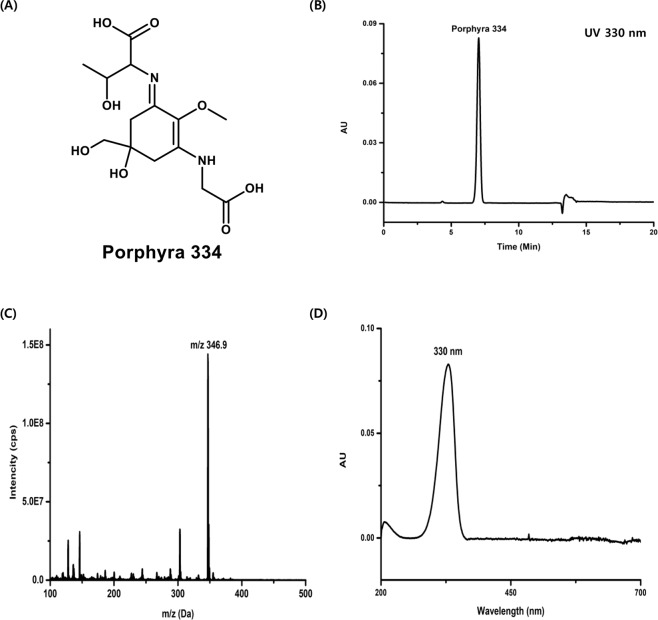
Characterization of P334. (**A**) The chemical structure of P334. (**B**) The result of HPLC analysis of P334. (**C**) The result of ESI-MS analysis of P334. (**D**) UV spectrum analysis of P334 dissolved in water.

The structure of P334 is shown in Fig. 1A. P334 was extracted from seaweed *Porphyra yezoensis* and dissolved in distilled water. To confirm the formation of P334, HPLC analysis was carried out. We observed a typical spectrum with prominent spectral peaks with a retention time of 7.04 min for P334, confirming that it was present in the mixture of purified MAAs (Fig. 1B). After purifying P334 from the mixture, the purity was measured with HPLC-Photo Diode Array Detector (Supplementary Fig. 1A). Several minor peaks were identified in the chromatogram as UV 330 nm, but the P334 peak area represented 99.63% of the total peak area (Supplementary Fig. 1B). Mass spectrometry (MS) is an invaluable technique for the detection of P334 and the identification of MAAs because of its high sensitivity. The protonated molecular ion [M + H]^+^ of P334 was at m/z 346.9, and the positive electrospray ionisation (ESI) mass spectrum showed distinctive features (Fig. 1C). Additionally, the UV-visible (UV-Vis) spectral analysis revealed a pronounced increase in absorption in the near-UV region at ~330 nm (Fig. 1D). The structure of P334 was confirmed by ^1^H and ^13^C nuclear magnetic resonance (NMR) at 700 and 175 MHz, respectively. ^1^H and ^13^C spectroscopic data and the positions of carbons are shown (Supplementary Fig. 2A,B).

### P334 accelerates cell reprogramming

**Figure 2:**
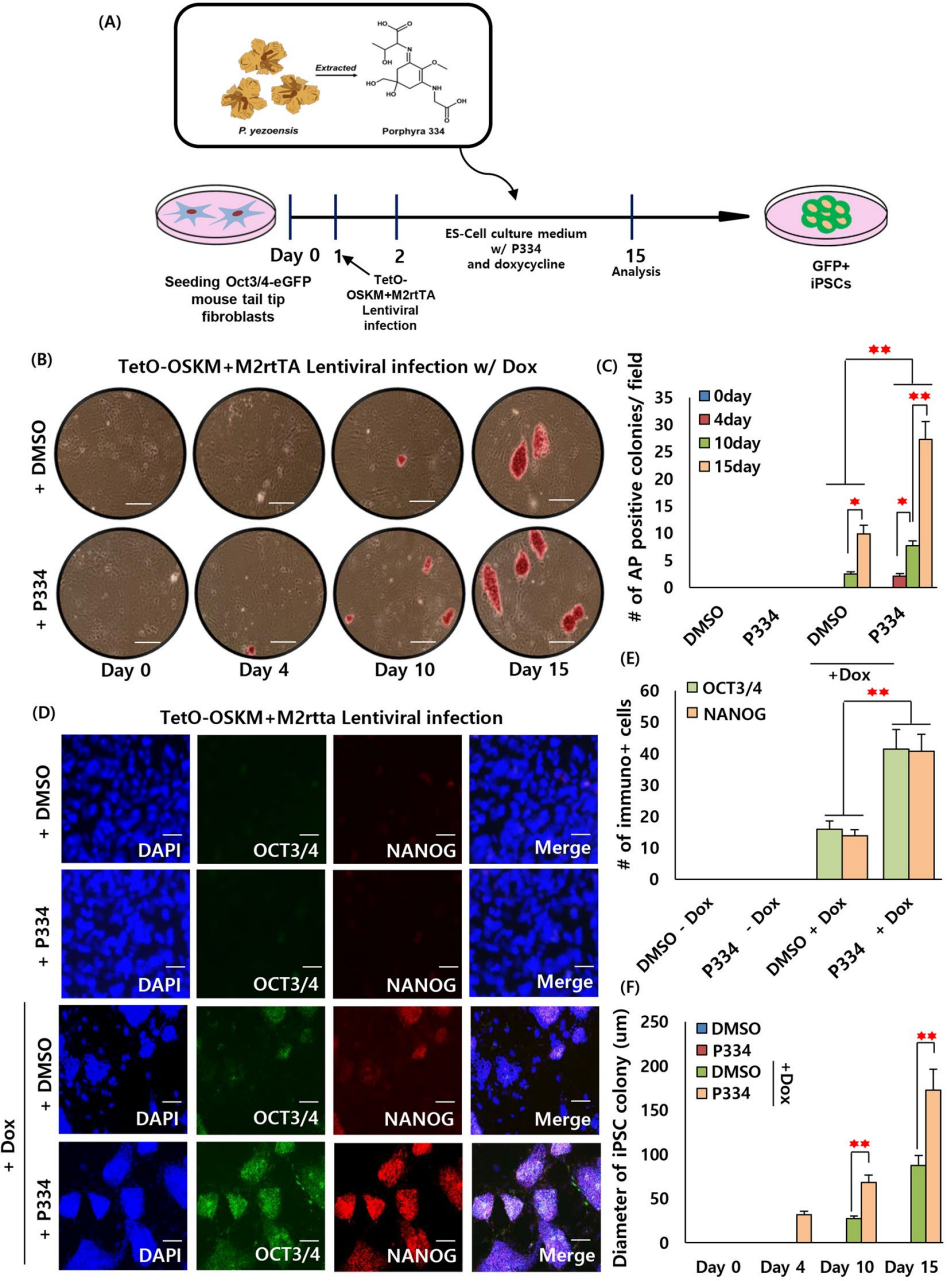
P334 mediates efficient generation of iPSCs from mouse somatic cells. (**A**) Schematic diagram describing the main theme and experimental process. (**B**) Representative AP (alkaline phosphatase) staining images of the generated iPSCs with and without P334 treated at 0 to 15 days after dox induction. Three independent experiments of three sets each were performed with 10 visual fields per set. (Scale bar: 200 um) (**C**) Bar graph showing the number of AP positive colonies with and without P334 treated at 0 to 15 days after dox induction (data represent mean ± SEM. *p < 0.05, **p < 0.01). (**D**) Immunostaining showing the expression of pluripotency markers, OCT3/4 and NANOG in each condition (control and P334 treated). (Scale bar: 100 um) (**E**) Bar graph representing number of OCT3/4 and NANOG positive cells per field (data represent mean ± SEM. **p < 0.01). (**F**) Bar graph demonstrating the size of iPSCs in each condition (control and P334 treated) (data represent mean ± SEM. **p < 0.01).

To determine whether P334 accelerates iPSC generation, Oct3/4-eGFP knock-in (KI) mouse tail-tip fibroblasts (TTFs) were prepared at a density of 4.3 × 10^5^ cells/cm^2^. One day after plating, TTFs were transduced using polycistronic doxycycline (dox)-inducible lentiviral vectors expressing the OSKM transcription factors along with a lentiviral vector constitutively expressing a modified reverse tetracycline trans-activator (M2rtTA). After viral infection, both dimethyl sulfoxide (DMSO) control-treated and P334-treated cultures were treated with dox. One day after viral infection we have switched the fibroblasts culture media to ESCs culture media (no vitamin C was used) (Fig. 2A).

**Figure 3:**
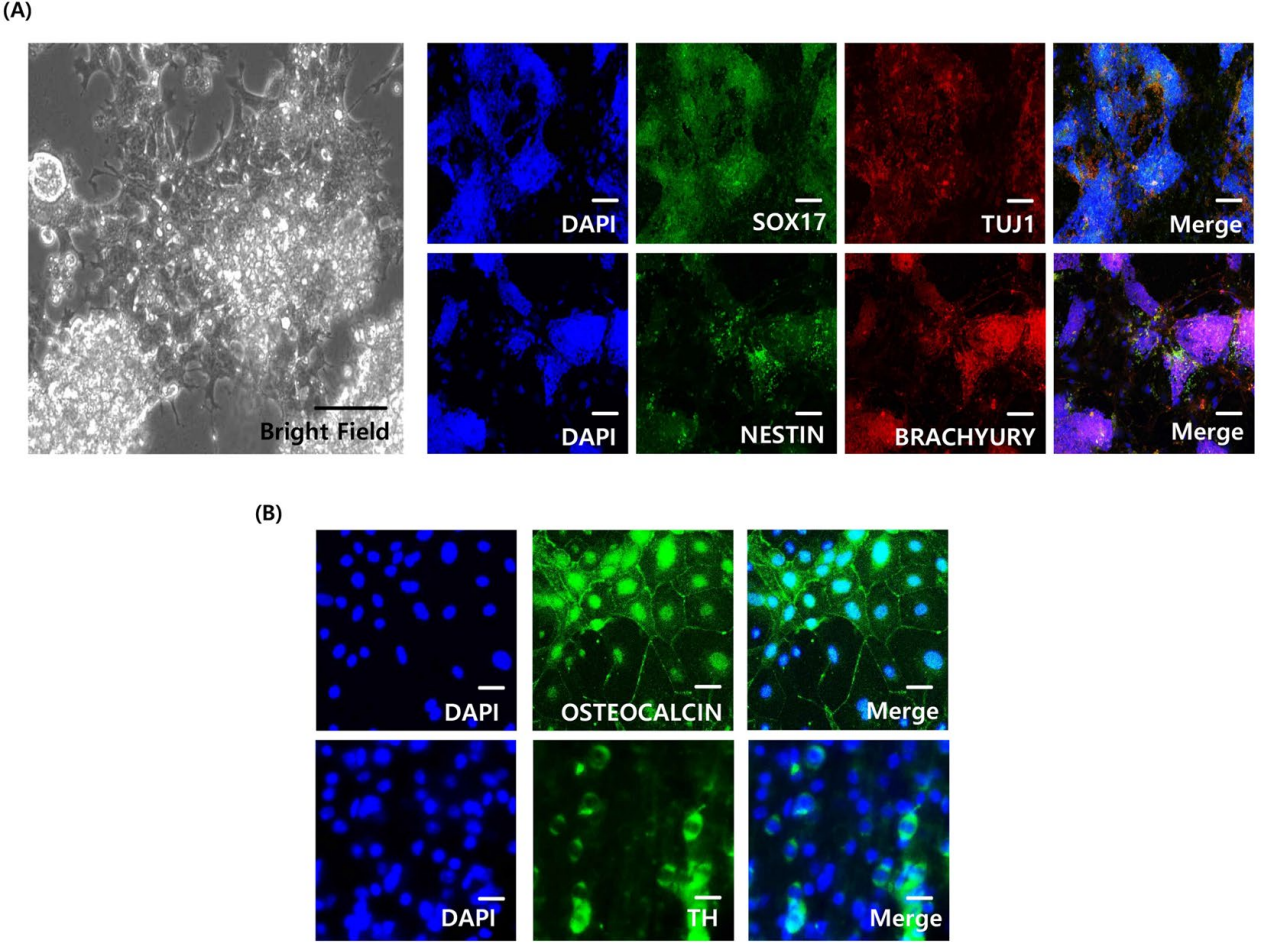
P334 mediated mouse iPSCs can be differentiated to three germ layers. (**A**) Mouse embryonic body (EB) based differentiation from P334 mediated iPS cells. Left panel showing the brightfield image of differentiated iPS cells. Right panel showing the immunostaining analysis of three germ makers in the differentiated P334 mediated iPS cells. Ectoderm: TUJ and NESTIN, Endoderm: SOX17 and Mesoderm: BRACHYURY. 15dpi iPSCs were used for EB generation and cultured extra 10 days after EB attachment process. (Scale bar: 100 um) (**B**) EB based differentiation from P334 mediated iPS cells to osteoblasts (marker: OSTEOCALCIN) and dopaminergic neuron (marker: TH). 15dpi iPSCs were used for differentiation and cultured extra 20 days after starting differentiation process. (Scale bar: 30 um).

We observed that TTFs began to undergo colony formation at 10 days (approximately 20 days in general) after viral infection in the P334-treated cultures and exhibited an increase in the number of colonies undergoing reprogramming, as assessed by morphology (Supplementary Fig. 3A,B). To quantify the reprogramming efficiency, we performed alkaline phosphatase (AP) staining after P334 treatment. In cells treated with P334, AP-positive iPSC colonies were measured 4 days after dox treatment. In contrast, the control group did not show AP-positive colonies until 10 days after dox induction. Consistent with this result, more AP-positive colonies were observed in the P334-treated group at 10 and 15 days after dox induction than in the control group (Fig. 2B,C). Additionally, we performed FACS analysis of Oct3/4-eGFP positive iPSCs derived from Oct3/4-eGFP KI TTFs in both the DMSO control group and the P334-treated group at 0 to 15 days after dox induction. Intriguingly, more GFP positive cells (approximately 5-fold more) were sorted in P334-treated culture at 4, 10 and 15 days after dox induction than in the control-treated culture (Supplementary Fig. 4A). Additionally, we confirmed that the P334-treated group exhibited a significant increase in the number of GFP positive cells (>5-fold higher), demonstrating that P334 facilitates cell reprogramming process with Yamanaka factors (OSKM) infection (Supplementary Fig. 4B,C)^18^. Additionally, the pluripotent state of P334-induced iPSCs was assessed by immunostaining analysis of pluripotency markers such as OCT3/4 and NANOG (Fig. 2D). Consistent with our hypothesis, we observed an increase in the number of OCT3/4 and NANOG positive colonies and in the size (iPSC colony diameter) of iPSCs in P334-treated cultures (Fig. 2E,F). Moreover, to determine the differentiation potential of P334-induced iPSCs, we tested their capacity to differentiate into cell types of the three germ layers. After 8 days of adherent culture, embryonic bodies (EBs) formed. EBs were placed in 6-well plates and were allowed to attach, and cell differentiation medium was used. Ten days after differentiation, we observed cells positive for Sox17 (endoderm marker), Tuj1, Nestin (ectoderm marker), and Brachyury (mesoderm marker) with immunostaining analysis (Fig. 3A). Additionally, we further verified the ability of P334-induced iPSCs to differentiate to osteoblasts and midbrain dopaminergic neurons. Twenty days after differentiation induction, we observed osteogenic and dopaminergic neuronal morphologies and confirmed the differentiation with immunostaining analysis (Fig. 3B).

### Mechanism of P334-induced cell reprogramming

**Figure 4:**
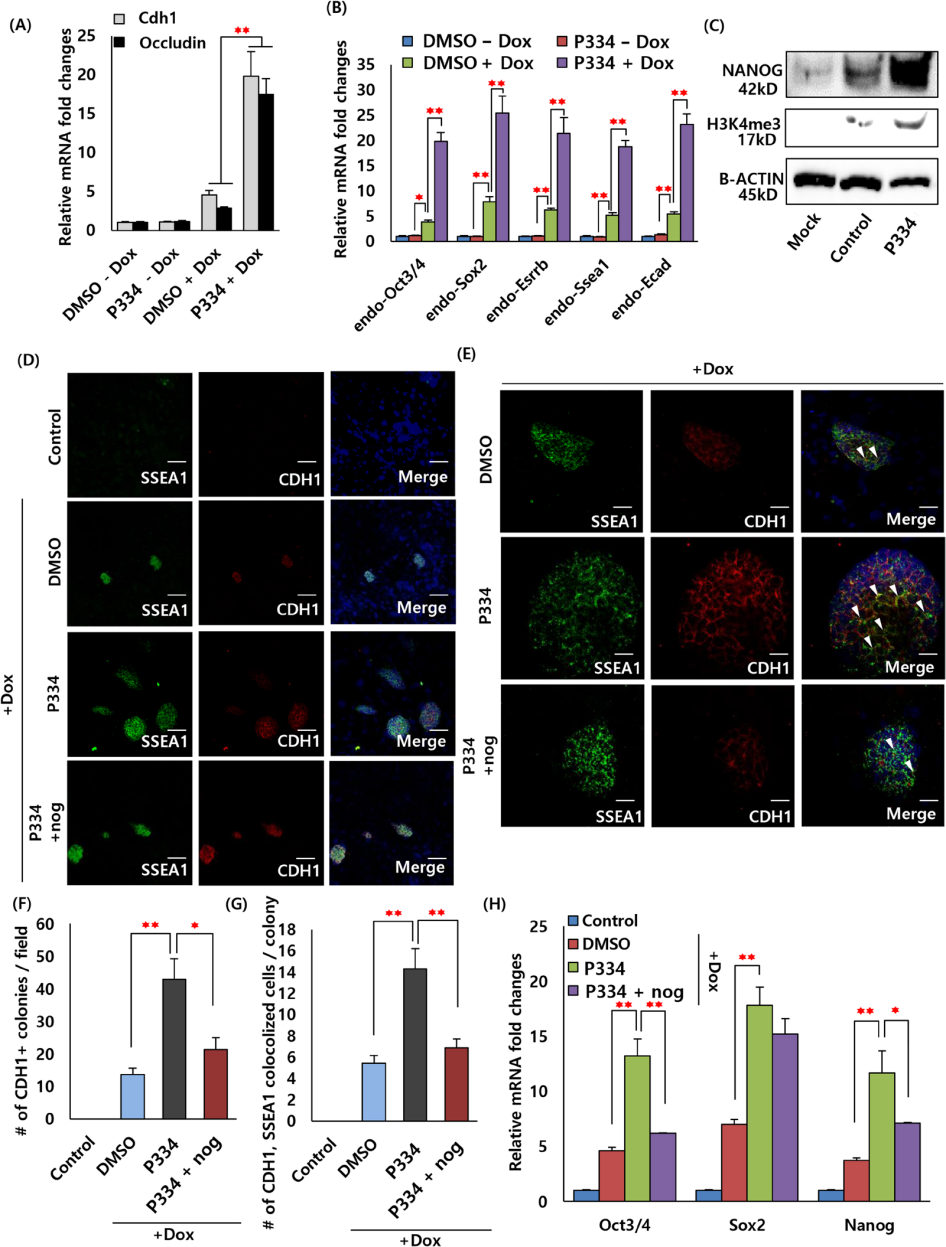
MET process with epigenetic changes associated with somatic cell reprogramming induced by P334. (**A**) RT-PCR analysis of MET genes following reprogramming in control and P334 treated after dox induction (data represent mean ± SEM. **p < 0.01). (**B**) RT-PCR analysis of pluripotent markers Oct3/4, Sox2, Esrrb, Ssea1 and E-cadherin (Cdh1) in OSKM-infected fibroblasts with and without P334 treated at 15 days after dox induction (data represent mean ± SEM. *p < 0.05, **p < 0.01). (**C**) Western blot analysis of histone 3 lysine 4 tri-methylation (H3K4me3) and NANOG of 4 factors infected fibroblasts reprogramming in control and P334 treated batch. (**D**) Immunostaining analysis showing the expression of pluripotency markers, SSEA1 and CDH1, in OSKM-infected fibroblasts with and without P334 and noggin (nog) treated at 15 days after dox treated. (Scale bar: 150 um) (**E**) Immunostaining analysis showing the co-localization of pluripotency markers, SSEA1 and CDH1, in OSKM-infected fibroblasts with and without P334 and nog treated at 15 days after dox treated. More amount of co-localized cells were observed in P334 treated batch. However, lowered amount of co-localized cells were measured in nog treated batch. (White triangle showing the co-localized cells in single iPSC colony.) (Scale bar: 30 um) (**F**) Bar graph representing the number of CDH1 positive iPSC colonies per field (data represent mean ± SEM. *p < 0.05, **p < 0.01). (**G**) Bar graph representing the number of co-localized CDH1 and SSEA1 positive cells in single iPSC colony (data represent mean ± SEM. **p < 0.01). (**H**) QRT-PCR analysis of pluripotency genes (Oct3/4, Sox2 and Nanog) in OSKM-infected fibroblasts with and without P334 and nog treated (data represent mean ± SEM. *p < 0.05, **p < 0.01). Data is analyzed at 15 days after dox induction.

Next, we determined how P334 influences the somatic cell reprogramming process. Previously, it has been reported that P334 induces the activation of FAK, which controls mesenchymal to epithelial transition (MET) and epithelial to mesenchymal transition (EMT). Several lines of evidence suggest that MET and EMT play key roles in the early and late stages of cell reprogramming into pluripotent lineages^21,28^. It has been shown that fibroblasts undergo MET during reprogramming, a process that is marked by the upregulation of epithelial markers such as Cdh1 and Occludin. Prior studies have demonstrated that biomaterial substrates such as graphene and nanotopographical substrates can directly affect the MET process during cell reprogramming^29,30^. Thus, to determine whether P334 helps to increase reprogramming efficiency via activating MET during the reprogramming process, we plated TTFs at equal densities with, treated them with P334 treating and measured the gene expression levels of MET-related markers (Cdh1 and Occludin) at 15 days after dox induction (Fig. 4A). We found that Cdh1, Occludin and pluripotency markers such as endogenous Oct3/4, Sox2, Esrrb, and Ssea1 were significantly upregulated in the P334-treated group (Fig. 4B). Since it was known that MET takes place in the early stage of reprogramming, we investigated the relative mRNA expression levels of Cdh1 at 0, 4, 7, 15 and 25 days after dox induction in the presence and absence of P334 (Supplementary Fig. 5A). We observed a significant difference between the DMSO control and P334 groups starting at 4 days after dox induction and found a remarkable difference (>3-fold) at 15 days after dox induction. Consistent with this result, we also measured more CDH1 and NANOG positive iPSC colonies (>3.5-fold) at 7 and 15 days after dox treatment in the P334 treatment group (Supplementary Fig. 5B).

We also examined EMT marker genes (Slug and N-cadherin) at 15 days after dox induction and did not find a significant difference in these mRNA levels between the two groups (Supplementary Fig. 6). Thus, we further concluded that the agonistic effect on the MET process was applied to the P334-treated condition during cell reprogramming, leading to the upregulation of epithelial lineage marker gene expression levels. To determine whether P334 affects the chromatin state during cell reprogramming via MET, we analysed the enrichment level of a histone modification that marks the active form H3K4me3 in the P334-treated group at 15 days after dox induction. Surprisingly, we found a dramatic increase in both H3K4me3 and NANOG protein expression in P334-treated cultures (Fig. 4C). To explore this finding in more in detail, we investigated the relative immunofluorescence intensity of H3K4me3 and H3K27me3 (used as a negative control) at 15 days after dox induction and found a significant increase in H3K4me3 levels in the P334-treated cells, suggesting that H3K4me3 is the key histone modification marker for P334-mediated cell reprogramming (Supplementary Fig. 7A,B).

In general, MET is well known to be induced by the activation of the BMP signalling cascade. The activation of the BMP pathway augments the MET process, leading to efficient reprogramming^29,31,32^. Because we hypothesized that P334 accelerates the MET process in this study, we treated the cells with 1.5 nM noggin (nog), a BMP antagonist, for 5 days after dox induction. Interestingly, we found that the number of SSEA1 and CDH1 immuno positive colonies was dramatically reduced in nog-treated cultures 15 days after dox induction. Moreover, more co-localized SSEA1 and CDH1 immuno positive cells were observed in the P334-treated cells, which decreased to the control level in nog-treated cells (Fig. 4D–G). To investigate whether nog affects RNA levels as well as protein levels, we performed qRT-PCR analysis and found that the mRNA levels of endogenous Oct3/4, Nanog, Cdh1 and Occludin were decreased in nog-treated conditions (Fig. 4H and Supplementary Fig. 8A,B).

Taken together, these results suggest that P334 specifically accelerates the MET process in cell reprogramming by controlling histone modification, specifically H3K4me3.

### Generation of human induced pluripotent stem cells (hiPSCs) with P334

**Figure 5:**
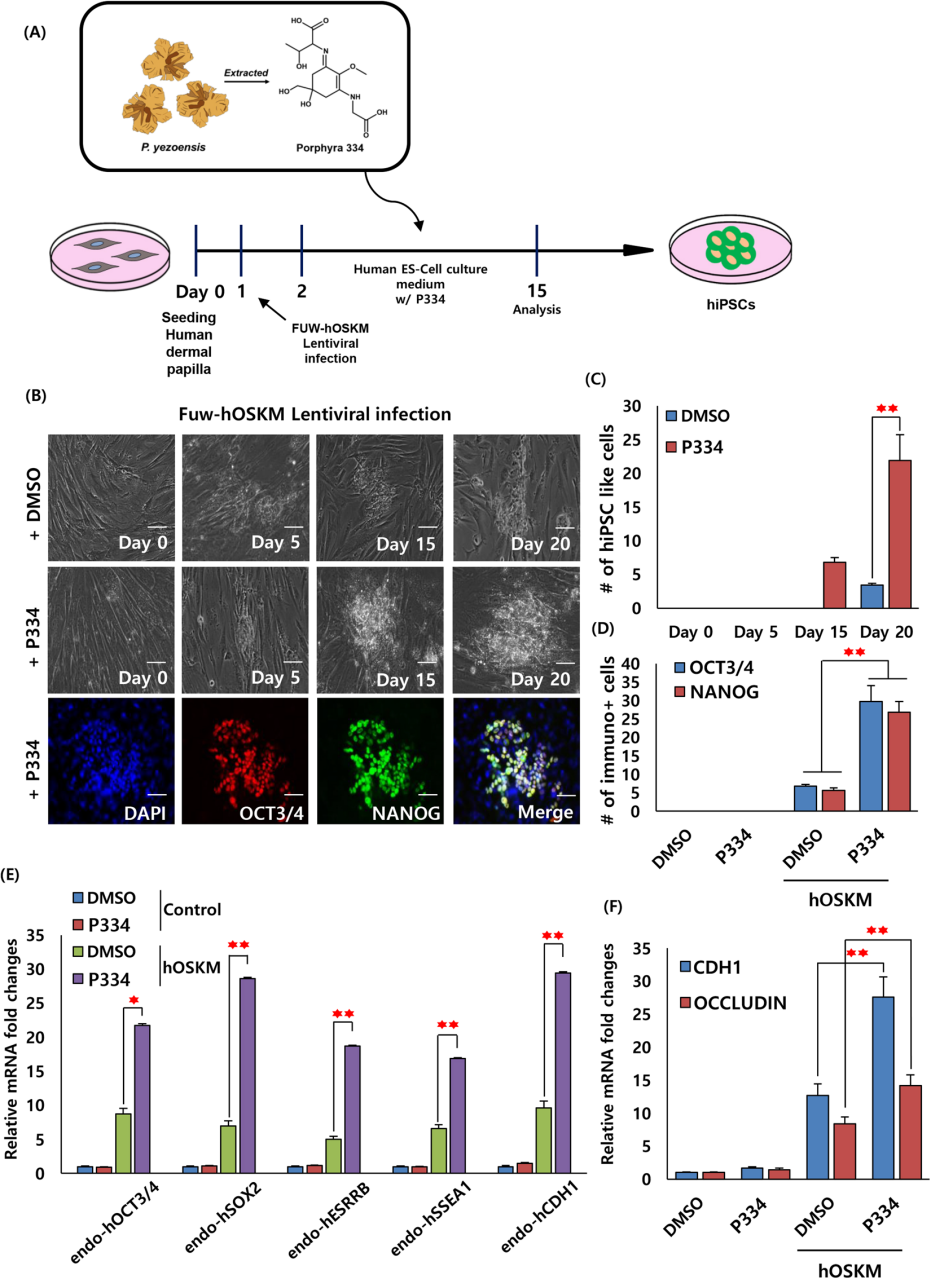
P334 accelerates generation of human iPSCs from human fibroblasts during the reprogramming process. (**A**) Schematic diagram describing the main theme and experimental procedure of human dermal papilla (HDP) reprogramming. (**B**) Representative images of the generated human iPSCs (0, 5, 15 and 20 days after lentivirus infection) in DMSO control and P334 treated. The bottom row representing the OCT3/4 and NANOG positive immuno fluorescence images in DMSO control and P334 treated batches. (Scale bar: 30 um) (**C**) The bar graph representing the number of human iPSCs in DMSO control and P334 treated batches at 0, 5, 15 and 20 days after lentivirus infection (data represent mean ± SEM. *p < 0.05, **p < 0.01). (**D**) The bar graph representing number of OCT3/4 and NANOG immuno positive colonies in DMSO control and P334 treated batches at 20 days after lentivirus infection (data represent mean ± SEM. **p < 0.01). (**E**) QRT-PCR analysis of pluripotent markers OCT3/4, SOX2, ESRRB, SSEA1 and CDH1 in hOSKM-infected HDP with and without P334 treated at 20 days after lentivirus infection (data represent mean ± SEM. *p < 0.05, **p < 0.01). (**F**) QRT-PCR analysis of MET genes (CDH1 and OCCLUDIN) in OSKM-infected HDP with P334 treated at 20 days after lentivirus infection (data represent mean ± SEM. **p < 0.01).

The marked cell reprogramming of mouse somatic cells with P334 prompted us to determine whether P334 would increase the reprogramming efficiency of human somatic fibroblasts into hiPSCs. To test this hypothesis, we prepared human dermal papilla (HDP) cells and transduced them with human fuw-Oct4-Sox2-Klf4-cMyc lentiviral vectors and treated them with P334. After viral infection, both the control- and P334-treated cultures were cultivated in ESC culture medium with FGF (Fig. 5A). Three weeks after induction, in the number of OCT3/4 and NANOG positive colonies increased in P334-treated cultures (Fig. 5B–D). The mRNA levels of pluripotency markers, such as endogenous OCT3/4, SOX2, ESRRB, SSEA1, and CDH1, were also significantly upregulated in the P334-treated cultures (Fig. 5E). As in mouse cells, MET-related genes were highly upregulated in P334-treated cultures, whereas for the upregulation of EMT-related genes was not observed (Fig. 5F and Supplementary Fig. 9). These results suggest that P334 triggers human somatic cell reprogramming by upregulating MET, thus promoting histone modification to facilitate somatic cell reprogramming.

## Conclusion and Discussion

**Figure 6:**
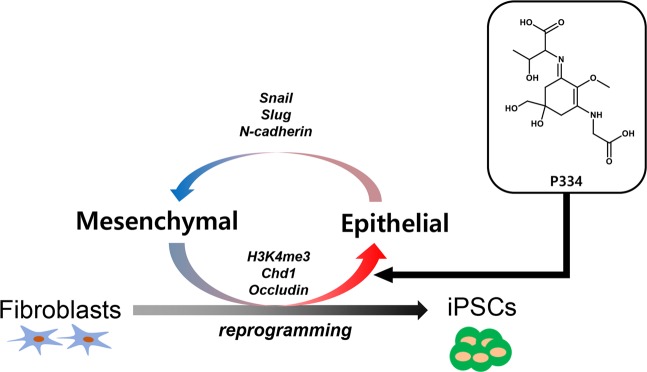
A summary of P334 inducing efficient cell reprogramming via MET.

In conclusion, we identified a novel role of P334 in somatic cell reprogramming. P334 serves as an excellent substrate for efficient cell reprogramming. We also determined that MET mediates P334-induced epigenetic changes during cell reprogramming (Fig. 6). Additionally, vitamin C (VitC), a nutrient known for its anti-oxidation activity, has also been shown to promote the generation of iPSCs from somatic cells through epigenetic modulation^33–36^. Therefore, we compared the reprogramming efficiency of P334-treated culture with VitC treated culture by immunostaining analysis. In both VitC and P334 treated cultures, we found more OCT3/4 and NANOG positive iPSC colonies than in control-treated cultures, showing that P334 plays an essential role in the cell reprogramming process, similar to VitC (Supplementary Fig. 10A,B). Taken together, our results support that P334 represents an excellent natural compound for promoting the cell fate conversion associated with reprogramming and thus may have various applications in stem cell-based regenerative medicine therapies.

## Materials and Methods

### Preparation of P334

*P. yezoensis* was cultivated and collected in the south coast, Wando area, of S. Korea. The dried *P. yezoensis* of 100 g was swelled in 20-fold volume of distilled water for 2 hrs, then extracted at 45 °C for 12 hrs. The extract was filtered through 200 mesh cartridge filters and freeze-dried, then 16 g of dried powder was obtained. The dried powder was dissolved in distilled water and sterilized in an autoclave. For the isolation and purification of P334, the solution was filtered and purified using preparative HPLC (Waters 2525 binary gradient module pump, Waters, USA). The P334 fraction was freeze-dried, then 400 mg of purified P334 powder was yielded. For the experiments, we tested 0 to 300 uM of P334 to cell reprogramming and found optimal concentration (100 uM) for efficient cell reprogramming

The purified P334 was analyzed by HPLC (1525 μ Binary HPLC pump with a 2996 PDA detector, Waters, USA). The analytical column was a Gemini® C18 (5 μm, 4.6 × 250 mm, Phenomenex, USA) and Shim-pack GIS C18 (5 μm, 4.6 × 250 mm, Shimadzu, Japan). The mobile phase was 0.1% trifluoroacetic acid in water and 0.1% trifluoroacetic acid in acetonitrile at a 1 mL/min flow rate.

For MS analysis, an ESI MS/MS system (AB SCIEX 3200 QTRAP MS/MS, Applied Biosystems, USA) was used. P334 was quantified in Q1 MS (Q1) mode in positive mode. NMR spectra of the purified P334 was recorded on an ADVANCE III 700 MHz NMR spectrometer (Bruker, Germany). The sample was dissolved in D_2_O and transferred to the NMR tube, then ^1^H NMR and ^13^C NMR spectra were measured with tetra-methylsilane as an internal standard.

### Cell culture

HEK293 cells were used for packaging the lentivirus. Cells were grown in fibroblast media (high-glucose DMEM [Invitrogen], 10% FBS [Hyclone], and 5% penicillin/streptomycin [Invitrogen]). These cells were co-transfected with the lentivirus construct, psPAX2, pMDG and tetO-OSKM/FUW-M2rtTA vectors using calcium phosphate co-precipitation. The cell culture medium was replaced 24 hrs after transfection, and viruses were harvested 72 hrs later. Mouse fibroblasts were transduced (60,000 cells) at passage 2 or 3 in 6 well culture dishes with the lentivirus. Infected mouse fibroblasts were cultured in mESC media with doxycycline (Dox) (2 µg/mL).

### Alkaline phosphatase staining

Alkaline phosphatase staining was performed using an Alkaline Phosphatase Substrate Kit (Millipore) according to the manufacturer’s recommendations. To determine the number of AP+ colonies, equal numbers of cells were plated on 100-mm dishes coated with gelatin. Experiments were repeated three times, and data represent the mean of triplicate wells ±SEM.

### Immunofluorescence analysis

iPS cells were cultured on 6 well cell culture plates and fixed with 4% PFA. The cells were then stained with primary antibodies against human/mouse Oct4 (Santa Cruz); mouse Nanog (Bethyl Lab), H3K4me3 (Abcam), and H3K27me3 (Millipore); and human/mouse Sox2 (R&D), Oct3/4, and Nanog (DSHB). Respective secondary antibodies were conjugated to Alexa Fluor (Invitrogen). Nuclei were counterstained with 4, 6-diamidino-2-phenylindole (DAPI; Invitrogen). Cells were imaged with a Nikon eclipse Ti microscope. Images were processed and analyzed with Adobe Photoshop software.

### Western blotting

Western blotting was carried out as described previously^37^. Rabbit polyclonal anti-H3K4me3 (Abcam), Nanog (Santa Cruz) and beta-actin (Cell signaling) antibody were used. Full length of western gel images for anti-Nanog, anti-H3k4me3 and anti-beta actin were presented in Supplementary Fig. 11.

### Flow cytometry

Flow cytometry was performed on a C6 cytometer (Accuri). Data were analyzed with FlowJo software (TreeStar). Briefly, cells were dissociated with trypsin for 5 minutes, and single cells were then pelleted, resuspended in ice-cold 4% paraformaldehyde, and incubated for 10 min at 4 °C. The cells were washed twice and resuspended in FACS buffer for FACS analysis.

### Quantitative real time-PCR

For quantitative PCR (qPCR) analysis, RNA was isolated using a PureLink RNA Mini Kit (Ambion). Complementary DNA was produced with the Super Script III Kit (Invitrogen). Real-time quantitative PCR reactions were set up in triplicate with the SYBR FAST qPCR Kit (KAPA) and run on a StepOnePlus Real-Time PCR system (Applied Biosystems). The housekeeping gene GAPDH, was used for data normalization. The ΔC_t_ values of the P334-treated cells were compared with those of the untreated cells. A previously described mouse primer sequence^18^ was used as the primer sequence. All primers were purchased from Cosmo-geneTECH.

### Statistical analysis

All data were analyzed using Student’s t test or ANOVA analysis. P values less than 0.05 were considered significant (*p < 0.05, **p < 0.01).

## Supplementary information

Supplementary figures.

## Data Availability

Data is available upon request.
