# Supplementary material for: Acceleration of somatic cell reprogramming into the induced pluripotent stem cell using a mycosporine-like amino acid, Porphyra 334
Source: Sci Rep. 2020 Feb 28;10:3684. doi: 10.1038/s41598-020-60680-5 (PMC7048830; doi:10.1038/s41598-020-60680-5)
Supplement: Supplementary file 1 — Supplementary figures. [file 41598_2020_60680_MOESM1_ESM.pdf]

Acceleration of somatic cell reprogramming into the induced pluripotent stem cell using a mycosporine-like amino acid, Porphyra 334

Junsang Yoo[1, 2#], Junyeop Kim[3#], Jeong Hun Lee[1], Hyein Kim[1], Sung Joo Jang[1], Hyo Hyun Seo[1], Seong Taek Oh[1], Seung Jae Hyeon[2], Hoon Ryu[2], Jongpil Kim[3], Sang Hyun Moh[1*]

[1] Anti-aging Research Institute, BIO-FD&C Co., Ltd, Inchon 21990, Korea

[2] Center for Neuroscience, Brain Science Institute, Korea Institute of Science and Technology, Seoul 02792, Korea

[3]Department of Biomedical Engineering, Dongguk University, Seoul 100-715, Korea

[#]Junsang Yoo and Junyeop Kim contributed equally to this work.

* Correspondence should be addressed to Sang Hyun Moh Ph.D.

* Correspondence to [biofdnc@gmail.com]

Supplementary figure1.

(A)

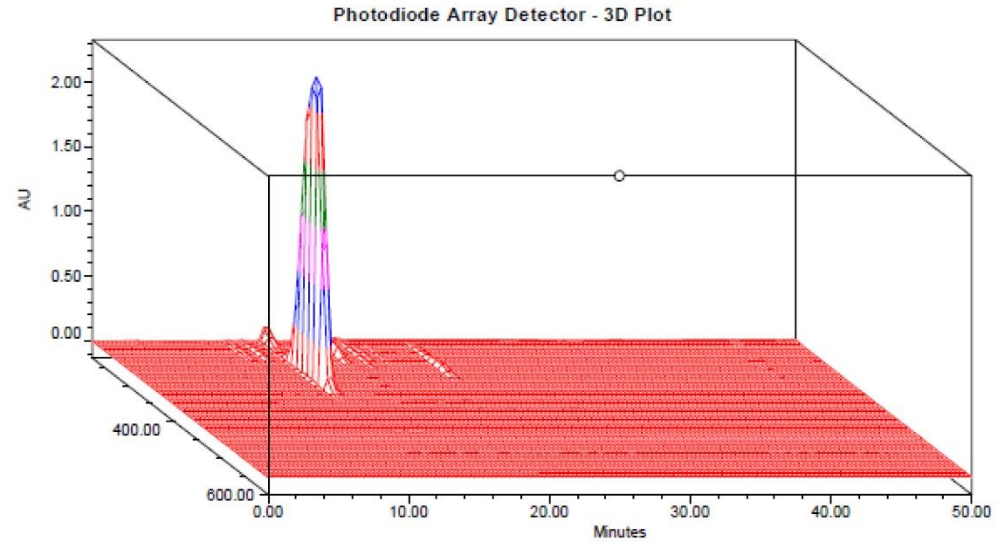

(B)

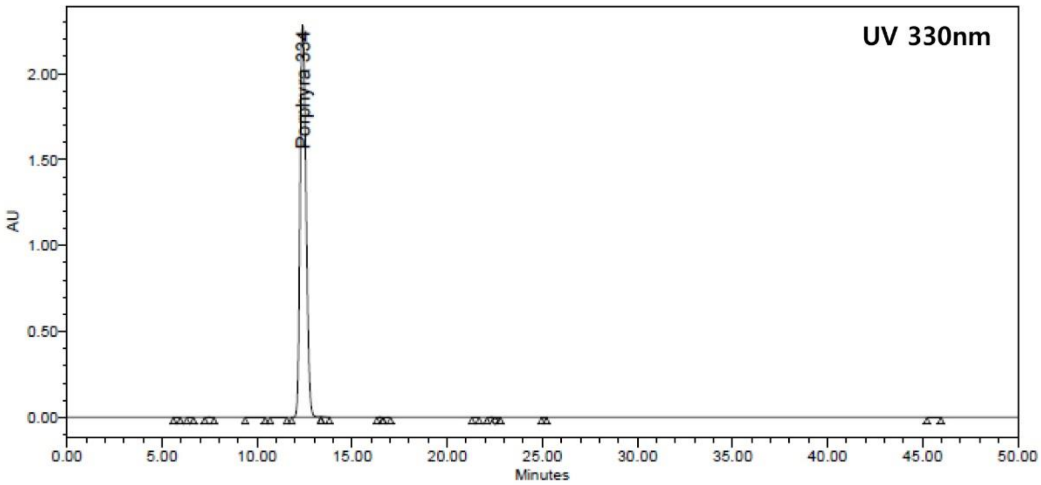

	Peak Name	RT	Area	% Area	Height
1		5.769	11079	0.02	1140
2		6.473	7482	0.01	755
3		7.477	33719	0.07	2842
4		9.988	20691	0.04	668
5		11.174	10358	0.02	418
6	Porphyra 334	12.392	49976862	99.63	2282742
7		13.550	9041	0.02	692

	Peak Name	RT	Area	% Area	Height
8		16.464	18470	0.04	2702
9		16.868	11464	0.02	1510
10		21.477	11161	0.02	1202
11		22.329	26499	0.05	2535
12		22.657	3818	0.01	461
13		25.085	2423	0.00	270
14		45.590	20159	0.04	1099

HPLC Chromatogram of P334 to confirm the purity A. The 3D plot of P334 measured using Photodiode Array Detector with Shim-pack GIS C18 column. B. The retention time of P334 was 12.392 min. The area ratio was 99.63% to the total peak area (at UV 330 nm).

Supplementary figure2.

(A)

Position	Porphyra 334	
	δH mult., (JHz)	δC mult.
1		160.2s
2		126.1s
3		161.6s
4	2.76d (17.5), 2.70d (17.5)	32.8t
5		71.0s
6	2.81d (17.5), 2.68d (17.5)	33.1t
7	3.47s	67.3t
8	3.59s	59.4q
9	4.19d (6.3)	44.8t
10		172.4s
11	4.34d (3.5)	62.1d
12		173.0s
13	4.37dd (6.3, 3.5)	67.5d
14	1.16d (6.3)	19.0q

(B)

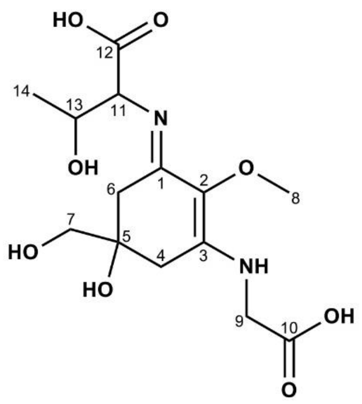

Porphyra 334

Identification of P334 structure by NMR spectroscopy A. ^{1}H and ^{13}C NMR spectroscopic data to confirm the structure of the purified P334. The sample was dissolved in D_2O with tetramethylsilane as an internal standard and measured at 700 and 175 MHz. B. The positions of carbons matched with each chemical shift (δH and δC).

Supplementary figure3.

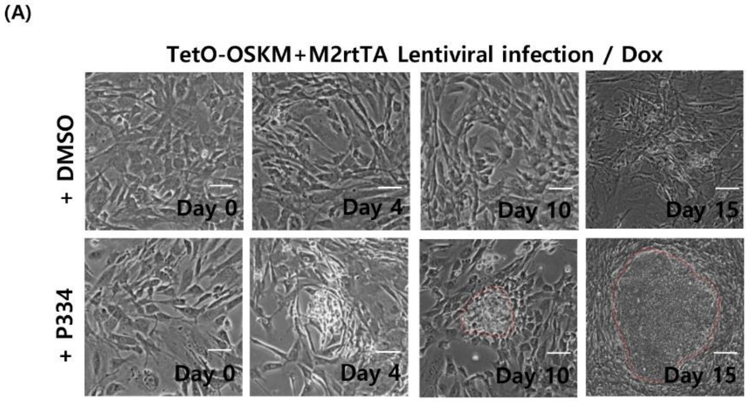

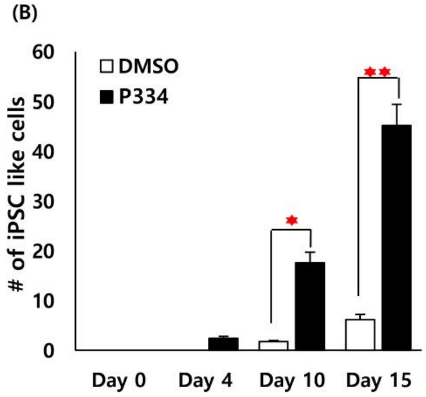

A. Representative bright field images of the generated iPSCs (0, 4, 10 and 15 days after lentivirus infection) in DMSO control and P334 treated batches. Scale bar, 100 ㎛. B. Bar graph showing the number of iPSCs in DMSO control and P334 treated batches (data represents mean ± SEM. *p < 0.05, **p < 0.01).

Supplementary figure4.

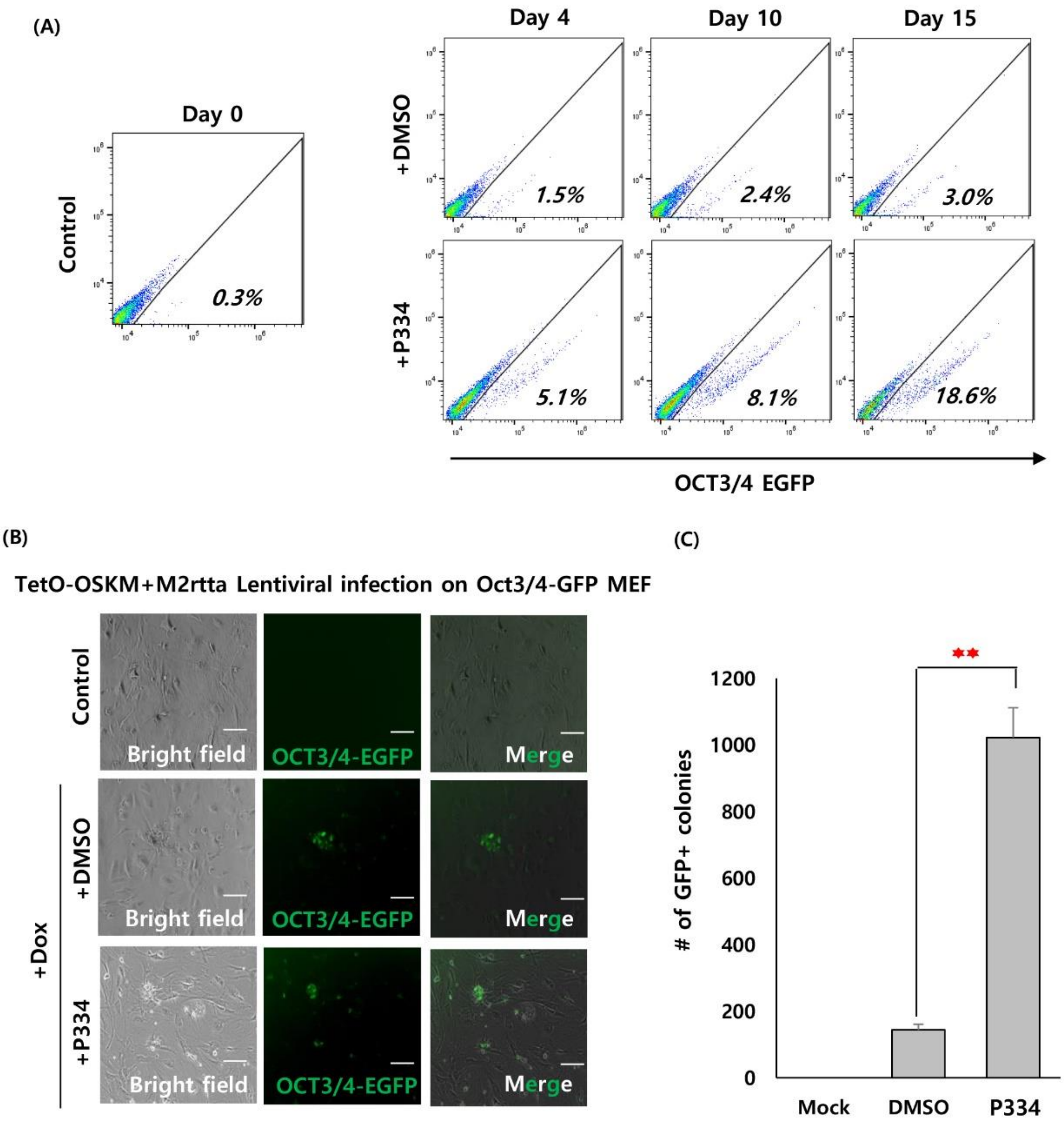

A. Fluorescence-activated cell sorting (FACS) analysis of Oct3/4-GFP knock in (KI) fibroblasts with and without P334 treated at 4, 10 and 15 days after dox induction. B. Endogenous GFP expression of iPSC colonies generated from Oct3/4-GFP knock in (KI) fibroblasts in DMSO control and the P334 treated batches. Scale bar, 150㎛. C. Bar graph representing the number of GFP positive colonies in DMSO control and the P334 treated batches (data represent mean ± SEM. **$p < 0.01$).

Supplementary figure5.

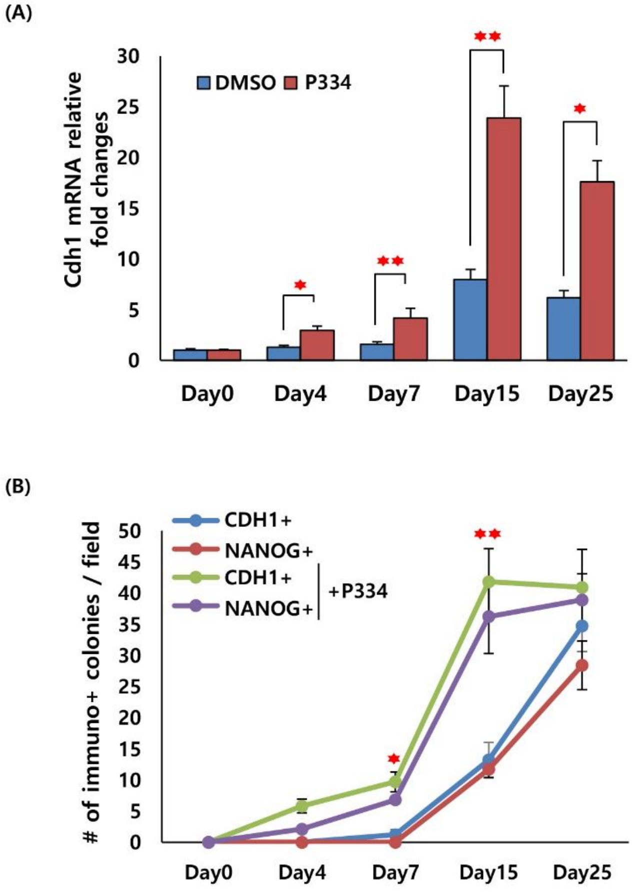

A. QRT-PCR analysis of Cdh1 in OSKM-infected fibroblasts with and without P334 (data represent mean ± SEM. *p < 0.05, **p < 0.01). Data is analyzed at 0 to 25 days after dox induction. B. Line graph demonstrating the number of CDH1 and NANOG immuno positive colonies with and without P334 treated at 4, 7, 15 and 25 days after dox induction.

Supplementary figure6.

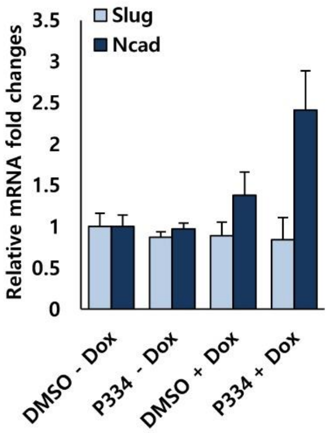

QRT-PCR analysis of EMT genes (Slug and N-cadherin) in OSKM-infected fibroblasts with and without P334. Data is analyzed at 15 days after dox induction.

Supplementary figure7.

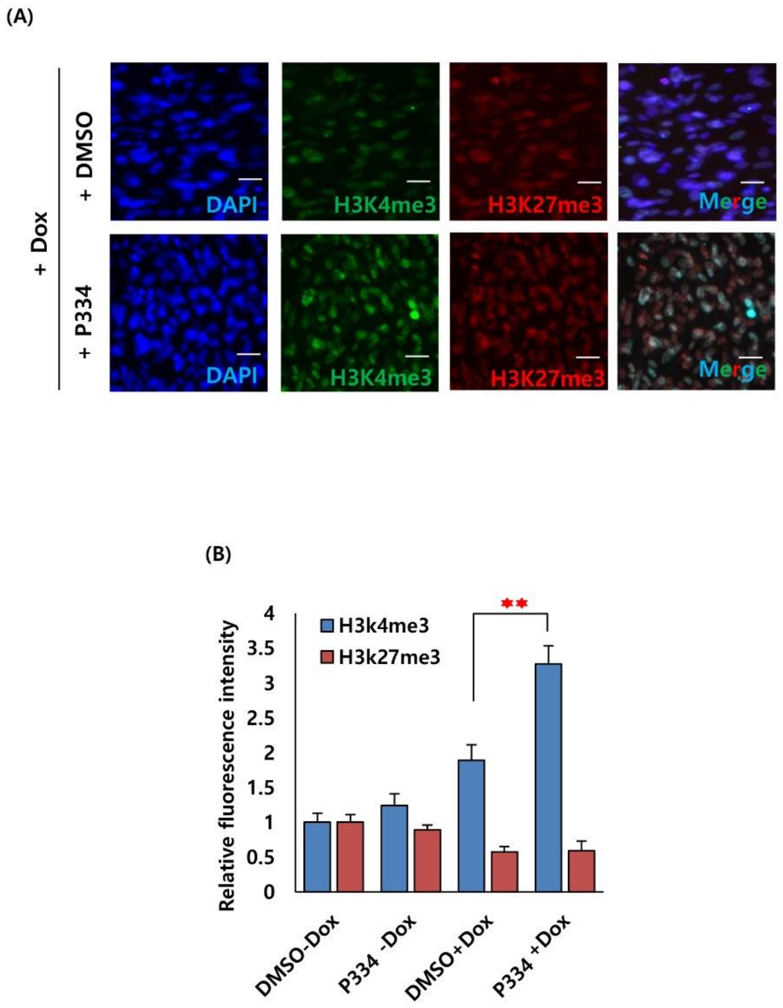

A. Immunostaining analysis of H3k4me3 and H3k27me3 in control and P334 treated at 15 days after dox induction. Scale bar, 100㎛. B. Bar graph representing the intensity of immuno fluorescence of H3K4me3 and H3K27me3 in control and P334 treated at 15 days after dox induction (data represent mean ± SEM. **$p < 0.01$).

Supplementary figure8.

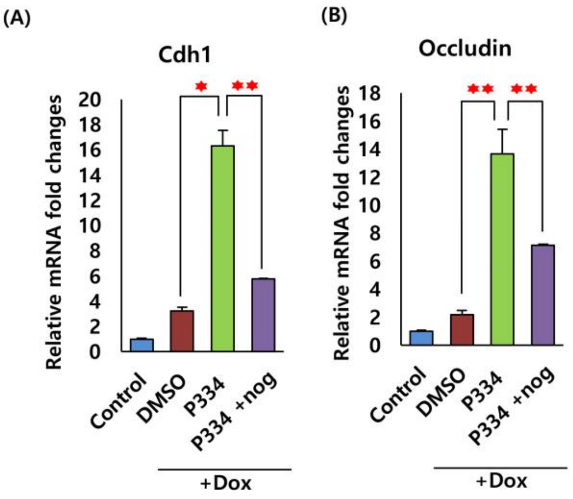

A. QRT-PCR analysis of MET genes (Cdh1 and Occludin) in OSKM-infected fibroblasts with and without P334 (data represent mean ± SEM. *p < 0.05, **p < 0.01). Data is analyzed at 15 days after dox induction. B. QRT-PCR analysis of EMT genes (Slug and N-cadherin) in OSKM-infected fibroblasts with and without P334. Data is analyzed at 15 days after dox induction (data represent mean ± SEM. **p < 0.01).

Supplementary figure9.

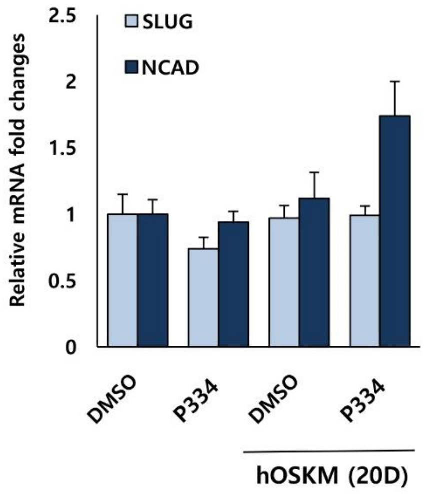

QRT-PCR analysis of EMT genes (Slug and N-cadherin) in human OSKM-infected fibroblasts with and without P334. Data is analyzed at 20 days after lentivirus infection.

Supplementary figure10.

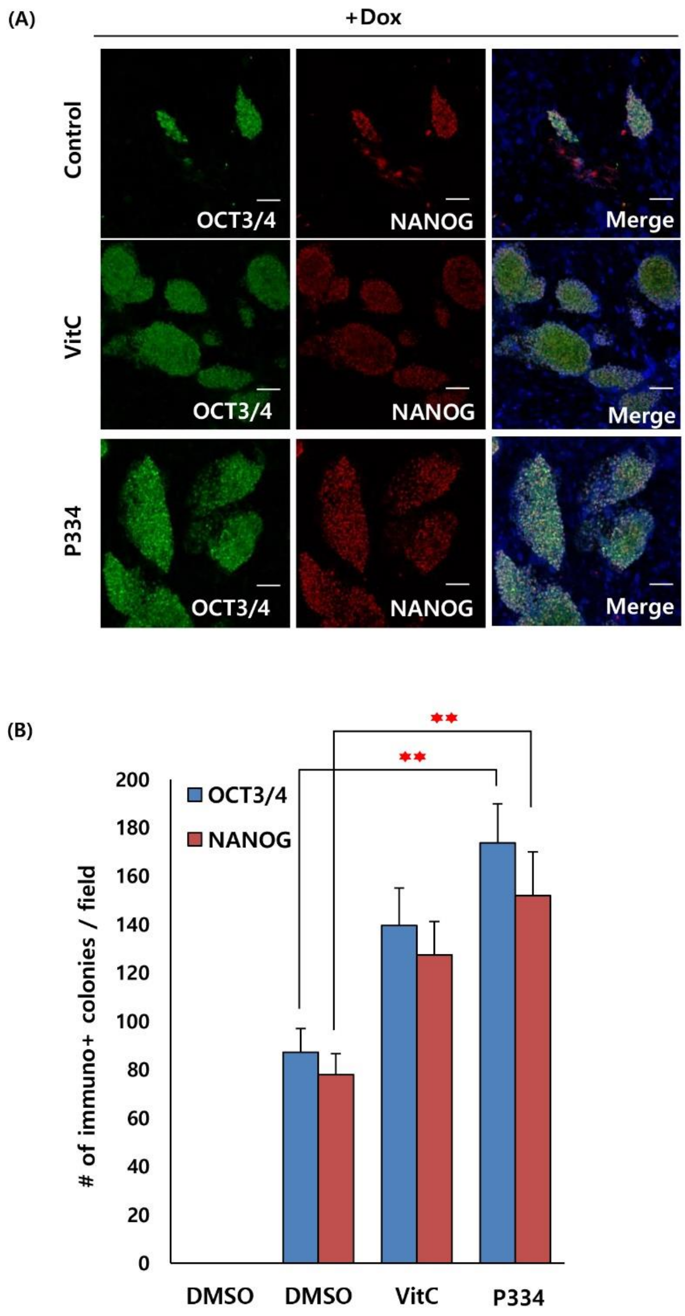

A. Immunostaining analysis of OCT3/4 and NANOG in control, Vitamin C and P334 treated at 15 days after dox induction. Scale bar, 200㎛. B. Bar graph representing the number of immuno positive colonies in control and P334 treated at 15 days after dox induction (data represent mean ± SEM. **$p < 0.01$).

Supplementary figure11.

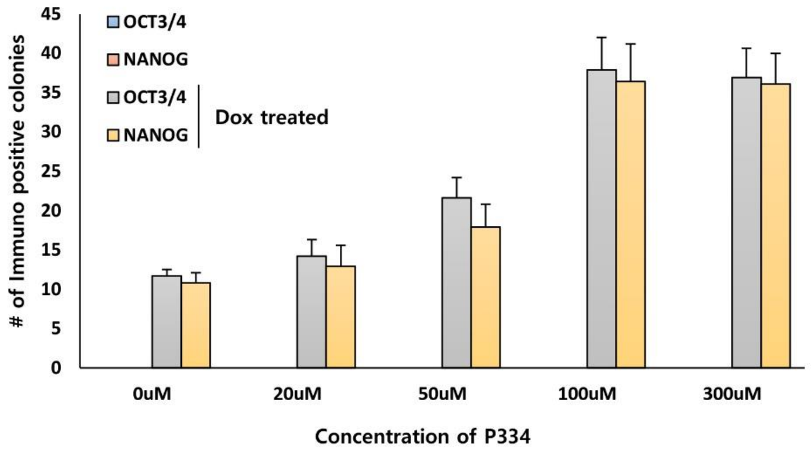

The bar graph representing number of immuno positive colonies under treatment of P334 dose dependently at 15 days after dox induction.

Supplementary figure12.

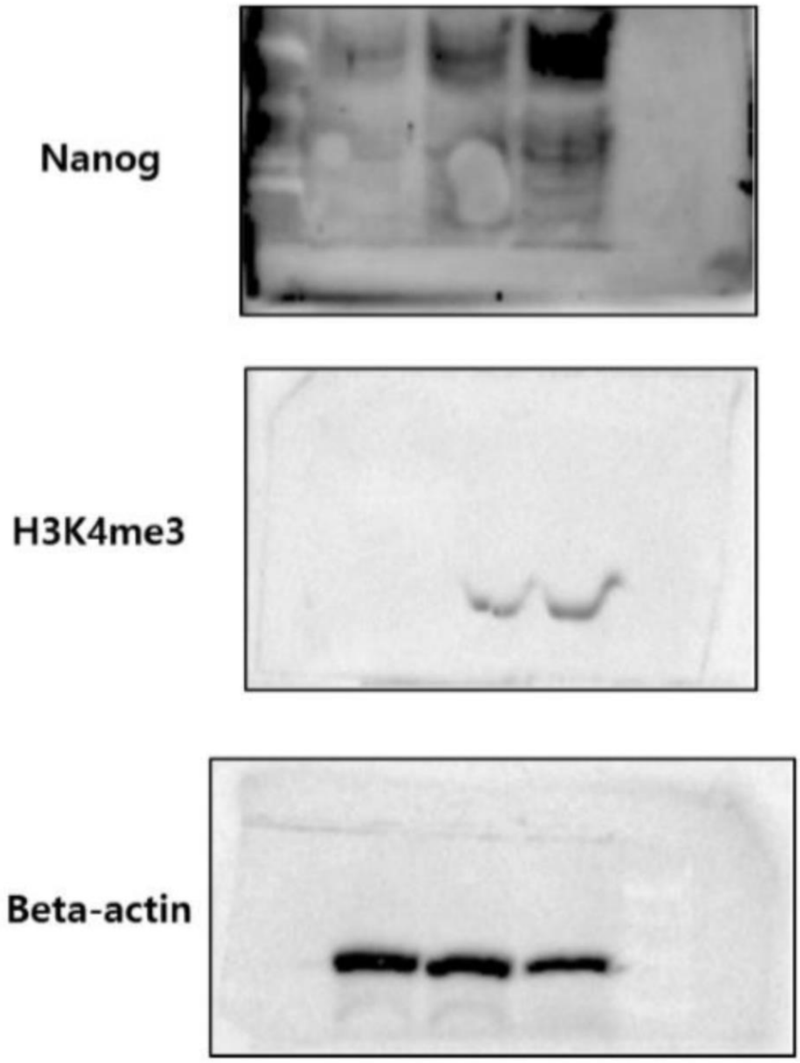

Full length western gel images for anti-Nanog, anti-H3k4me3 and anti-beta actin. (Nanog: Santa Cruz, 1E6C4; H3K4me3: abcam, ab8580; beta-actin: Cell Signaling)
